# Genome sequencing and assembly for *Aspergillus ustus* VKPM-F2030

**DOI:** 10.1128/mra.00697-26

**Published:** 2026-09-03

**Authors:** Elizaveta Popova, Anna Shestakova, Viktoriia Lavrenova, Evgenii Shelukhanov

**Affiliations:** 1 Department of Microbiology, Faculty of Biology, Lomonosov Moscow State University 64935, Moscow, Russia; University of California Riverside, Riverside, California, USA

**Keywords:** *Aspergillus*, fungi, proteases

## Abstract

The genome of *Aspergillus ustus* VKPM-F2030 strain was resequenced and assembled to achieve better quality and consistency of the assembly. It is the largest known genome of *Aspergillus* section *Usti* corresponding to 42 Mb and 25 contigs. This work can serve for further genomic investigations of *Aspergillus* fungi.

## ANNOUNCEMENT

*Aspergillus* fungi are ubiquitous, and representatives of the section *Usti* can exhibit different lifestyles and have large and rich genomes. A comparative genomic analysis within the section has been done by Nybo et al. in 2025, but this research has not included data on *Aspergillus ustus*, a fungus that gave the section its name (1). In 2013, *A. ustus* was isolated from an air sample of Lomonosov MSU (GenBank ID SAMN40546144). We previously demonstrated its capacity to produce diverse proteolytic enzymes with activity against fibrous proteins (2, 3). In 2024, we sequenced its genome using Illumina short reads (PRJNA1089602, GCA_040285355.1). The strain was deposited in the National Collection of Industrial Microorganisms (Russia). It is now referred to as *A. ustus* VKPM F-2030. To achieve better assembly quality, PacBio long-read sequencing was additionally performed.

**Fig 1:**
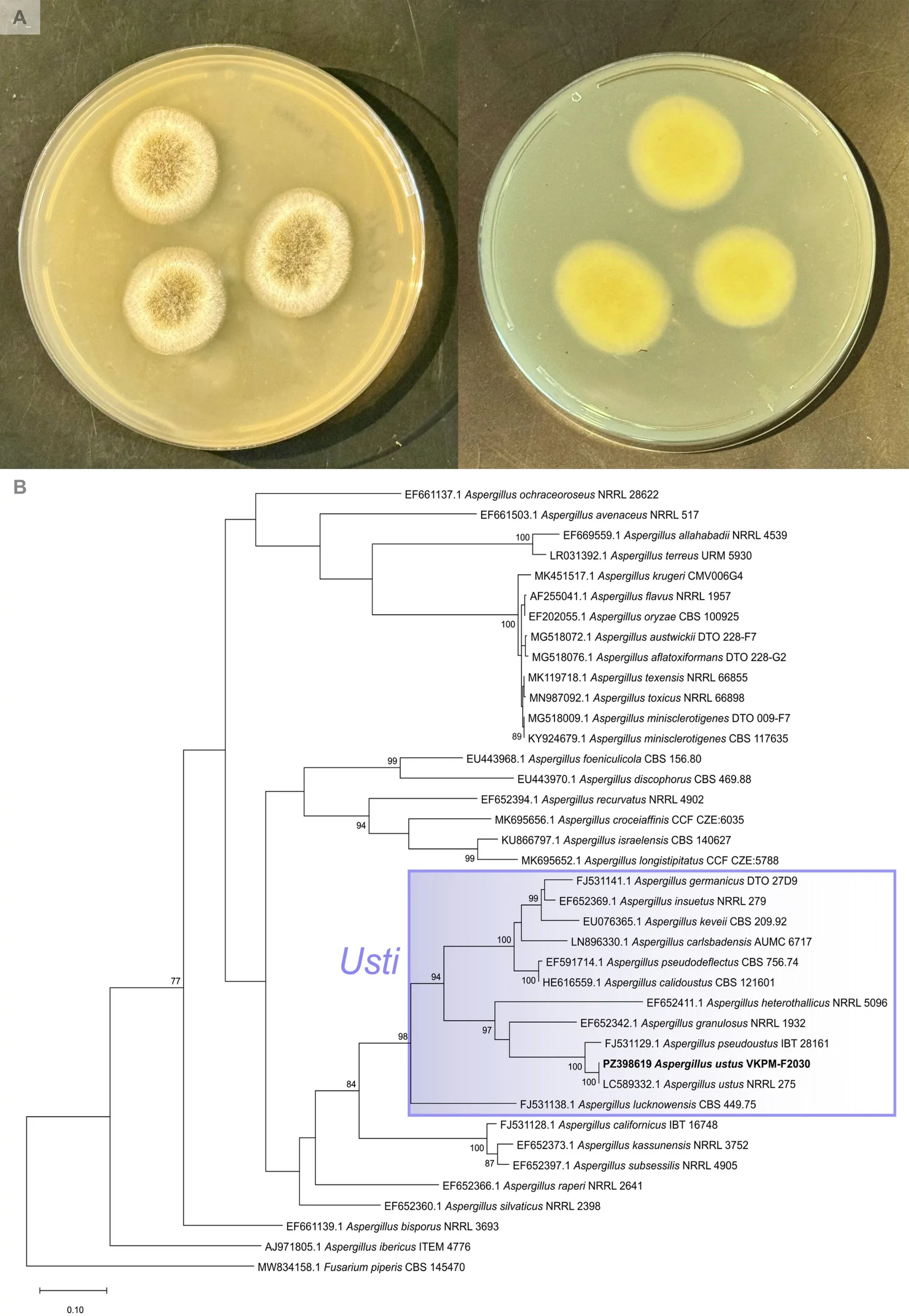
(**A**) *A. ustus* VKPM-F2030 grown on MEA for 5 days; (**B**) phylogenetic tree based on the *CaM* coding sequence.

*A. ustus* VKPM-F2030 cultivated on MEA at 28°C for 5 days (Fig. 1A) was used for biomass collection and DNA isolation (Quick-DNA Fungal/Bacterial Kit, Zymo Research). For PacBio sequencing, libraries were prepared with SMRTbell Prep Kit 3.0, and its concentration and size were measured with the Qubit HS DNA Kit (Invitrogen) and fragment analyzer (no shearing). Sequencing was conducted on the PacBio Revio System. Demultiplexing of the sequencing reads was performed with PacBio lima (2.5.1). HiFi reads (with a predicted accuracy ≥Q20 and no adapter trimming) were extracted using SAMtools (1.13) (4) and converted to FASTQ with PacBio bam2fastq (1.3.1) (5). For Illumina sequencing, DNA samples were used to create libraries using Illumina DNA Prep (M) Tagmentation (100 ng). Whole-genome sequencing (Illumina Novaseq Technology XPlus) was performed using the manufacturer’s protocol with a read length of 2 × 151 bp. Demultiplexing of sequencing reads was performed using Illumina bcl2fastq (2.20) (6). Adapters were trimmed using Skewer software (version 0.2.2) (7). The quality of FASTQ files was analyzed using FastQC software (version 0.11.5-cegat) (8).

Long reads were used to generate a primary assembly with NextDenovo (2.5.2) (9) and subsequently polished with short reads using the MaSuRCA pipeline (4.1.4) (10). Assembly quality was assessed using QUAST (5.3.0) (11), and genome completeness was estimated using BUSCO5 with an aspergillus-specific data set (12). A maximum-likelihood tree was built using MEGA11 based on the alignment of 38 *CaM* genes from different *Aspergillus* species (13). A calmodulin coding sequence was extracted during BUSCO analysis (contig 3, 3,695,907–3,696,633 b.). Default parameters were used, except where otherwise noted.

A total of 30,026,106 pair-end reads were generated with Illumina (4.4 Gbp) when PacBio sequencing resulted in 2,593,826 reads (1.6 Gbp) with a length distribution from 83 to 26,793 bp with a mode of approximately 4,500 bp. The final assembly consisted of 25 contigs with a total length of 42,066,436 bases and 50.83% guanine–cytosine (GC) content. Technical coverage was calculated as 142, and the final corresponded to 128. The largest contig was 8,662,253 bases. Assembly contiguity was high, with an N50 of 6,256,213 bases and an L50 of 3. BUSCO-assessed genome completeness corresponded to 97.2%.

Phylogenetic tree illustrating the taxonomy of *A. ustus* VKPM-F2030 is shown in Fig. 1B.

## Data Availability

This Whole-Genome Shotgun project has been deposited at DDBJ/ENA/GenBank under accession JBCHKC000000000. The version described in this paper is version JBCHKC020000000. Raw reads are available in Sequence Read Archive (SRA) under number PRJNA1089602. *CaM* gene of *A. ustus* VKPM-F2030 may be found using number PZ398619 in GenBank.
